# FASTNN: A Deep Learning Approach for Traffic Flow Prediction Considering Spatiotemporal Features

**DOI:** 10.3390/s22186921

**Published:** 2022-09-13

**Authors:** Qianqian Zhou, Nan Chen, Siwei Lin

**Affiliations:** 1 College of Computer and Data Science, Fuzhou University, Fuzhou 350108, China; 2 Key Laboratory of Spatial Data Mining & Information Sharing, Ministry of Education, Fuzhou 350108, China; 3 The Academy of Digital China (Fujian), Fuzhou University, Fuzhou 350108, China; 4 School of Geography and Ocean Science, Nanjing University, Nanjing 210023, China

**Keywords:** traffic flow prediction, spatiotemporal neural networks, spatiotemporal aggregation, filter spatial attention, matrix factorization based resample

## Abstract

Traffic flow forecasting is a critical input to intelligent transportation systems. Accurate traffic flow forecasting can provide an effective reference for implementing traffic management strategies, developing travel route planning, and public transportation risk assessment. Recent deep learning approaches of spatiotemporal neural networks to predict traffic flow show promise, but could be difficult to separately model the spatiotemporal aggregation in traffic data and intrinsic correlation or redundancy of spatiotemporal features extracted by the filter of the convolutional network. This can introduce biases in the predictions that interfere with subsequent planning decisions in transportation. To solve the mentioned problem, the filter attention-based spatiotemporal neural network (FASTNN) was proposed in this paper. First, the model used 3-dimensional convolutional neural networks to extract universal spatiotemporal dependencies from three types of historical traffic flow, the residual units were employed to prevent network degradation. Then, the filter spatial attention module was constructed to quantify the spatiotemporal aggregation of the features, thus enabling dynamic adjustment of the spatial weights. To model the intrinsic correlation and redundancy of features, this paper also constructed a lightweight module, named matrix factorization based resample module, which automatically learned the intrinsic correlation of the same features to enhance the concentration of the model on information-rich features, and used matrix factorization to reduce the redundant information between different features. The FASTNN has experimented on two large-scale real datasets (TaxiBJ and BikeNYC), and the experimental results show that the FASTNN has better prediction performance than various baselines and variant models.

## 1. Introduction

Intelligent transportation system (ITS) is a critical input to the development of transportation systems. It can effectively integrate advanced information and communication technologies to form a real-time, accurate, and efficient traffic management system [1,2,3,4]. Traffic flow prediction (TFP) is an important part component of ITS [5,6,7], whose objective is to predict short-term or long-term traffic flow based on historical traffic data (e.g., traffic flow, vehicle speed, etc.). In terms of traffic flow forecasting applications, take for example the more passenger-centric transportation systems of recent years, namely mobility on demand (MoD), which allows passengers to proactively submit travel requests specifying their pick-up and drop-off locations. However, the negative side of this transportation system is that if there is an imbalance between vehicle supply and order requests in a region, the system may have to allocate a distant vehicle to meet passenger travel demand, and passenger waiting time increases [8,9]. If the above occurs during peak periods or in a congested area, it may increase the travel burden in that area. Therefore, TFP for the region can pre-allocate the required vehicles to areas with high travel demand, which not only reduces passenger waiting time and improves travel service quality, but also provide references for implementing traffic management strategies, developing travel route planning, and public transportation risk assessment.

The key issue to achieving accurate predictions is modeling the high-dimensional and nonlinear spatiotemporal (ST) dependencies of massive traffic ST big data. Initially, researchers applied traditional machine learning methods for predictions, such as the ARIMA model [10], SVM [11], and SVR [12] models, etc. However, these models cannot effectively extract the ST dependencies between multi-source traffic data given their invariable model structures and weights. Moreover, the label features of machine learning models are dependent on intensive manual analysis, which also increases the subjective error of prediction results. Deep learning methods overcome these disadvantages through stacking neural network infrastructure and training the network with gradient descent [13]. It can realize automatic extraction of diverse ST dependencies by designing various neural networks. Thus, how to design the corresponding neural network to capture its complex spatial dependence and temporal dynamics is a current research hotspot. Zhang [14] extracted the ST features of the data based on deep neural networks; Niu [15] modeled the spatial dependence of the traffic data using convolutional neural networks (CNN) and long short-term memory (LSTM); Saxena [16] employed generative adversarial networks to model the multimodality of the data; Wang [17] used attention mechanisms to model the local and global temporal dynamics. Guo [18] captures the ST correlation and heterogeneity through 3D convolutional neural networks. While these methods have shown promise in improving TFP accuracy, it appears less capable of modeling ST aggregation and quantifying intrinsic correlation and redundancy of ST features.

To solve the mentioned problems, this paper proposed a deep learning-based ST prediction network model for predicting traffic flow, named the filter attention-based spatiotemporal neural network (FASTNN), which can sufficiently model the ST agglomeration of data, automatically learn the intrinsic correlation of ST features, and reduce the redundancy among diverse ST features. Specifically, based on 3D CNN and the residual unit, this paper proposed a filter spatial attention module (FSA) to model the ST agglomeration and dynamically adjust the region weights of each ST feature. Second, the matrix factorization based resample module (MFR) was proposed to automatically learn the intrinsic correlation of the same ST feature, and this module also reduces the redundant information contained between different ST features. Finally, this paper experimented with the FASTNN on two large-scale real datasets, including Taxi trip data in Beijing and bike-sharing data in New York, and the performance results with the baseline show the effectiveness of the FASTNN. The contributions of this paper can be summarized as follows:

(1) This paper proposed a traffic flow prediction model based on a deep learning framework, the FASTNN, which can model ST aggregation and quantify intrinsic correlation and redundancy of ST features.
(2) In this paper, filter spatial attention (FSA) was proposed to model the ST agglomeration of traffic data, and this module can implement dynamic adjustment of spatial weights.
(3) This paper proposed a lightweight module, the matrix factorization based resample module (MFR), which can model the intrinsic correlation of the same ST feature and reduce the redundant information between different ST features.

In the next section, the paper reviewed the existing literature on TFP and attention mechanisms in TFP research. Section 3 introduced the key concepts of the ST agglomeration and intrinsic correlation of the same ST feature, and also described the definitions related to TFP in this paper. In Section 4, the paper presented the framework of the proposed FASTNN model and the structure of the various components in the FASTNN. Experimental data and results were presented in Section 5. Finally, the conclusions were discussed in Section 6.

## 2. Related Works

Future traffic information is critical for MoD systems to improve their service quality and for policymakers to conduct effective transportation planning. Many researchers have investigated the related TFP problem [19]. TFP not only balances the supply and demand of future travel demand but also improves the operational efficiency of public transportation by formulating effective travel strategies based on the forecasted traffic information.

### 2.1. Traffic Flow Prediction

Traffic flow prediction (TFP) is a key problem in the field of data mining in urban computing [15,16]. Early TFP models were mainly based on statistical (e.g., autoregressive integrated moving average (ARIMA) [20], vector autoregression [21], etc.) or machine learning-based methods (including K-nearest neighbors [22], support vector machines [23], vector autoregressive moving average [24], etc.). For example, to address the sparsity and travel time uncertainty of real-time traffic data, Zhang [25] used the gradient-boosted regression tree method to extract the ST correlation of neighboring and target links of the road network to achieve temporal prediction of traffic flow; Cheng [26] proposed a multi-view learning algorithm for short-term traffic flow prediction, which can account for the temporal fluctuations and patterns of traffic in addition to the general spatial characteristics; Zhang [27] implemented a linear model with coefficients varying as a smoothing function of departure time to predict short-time travel times. However, it is difficult to extract the complex patterns hidden in the traffic flow because the above models have limited capacity to model complex traffic relationships nonlinearly. The scarcity of autonomous ability to learn combinational embeddings of ST features also represents a major challenge to these model-based approaches.

Given the complexity and variability of the actual traffic situation, the prediction accuracy of such models in the actual application requires to be improved to meet the requirements of ITS. To improve the model performance and achieve the depth extraction of ST dependencies, deep learning techniques with powerful feature extraction and non-linear fitting capabilities were widely accepted in TFP research. In time-dependent mining, Wu [28] used a Wave Net based on a one-dimensional temporal convolutional neural network to model the temporal correlation in traffic data; Fu [29] predicted traffic flow with LSTM and GRU networks; He [30] applied the sequence to sequence architecture to model the similarity of historical traffic flow between multiple time steps; To solve the subway traffic prediction problem, Liu [31] improved the LSTM network by using exogenous data, features of subway data, and temporal correlation; Du [32] proposed a deep irregular convolutional residual LSTM network model for urban traffic flow prediction to handle mixed traffic routes, mixed traffic, interchange stations, and some extreme weather; To predict the traffic congestion status of cities, Zhang [33] proposed a deep autoencoder neural networks to efficiently learn the temporal correlation of traffic networks.

The TFP deep learning method for mining time-dependent features has fewer training parameters and is highly efficient. However, the accuracy results of the prediction task for ST data still require to be improved because of its own inability to model the spatial correlation in the data. In spatial-dependent mining, TFP generally presents traffic data in a grid or pixel form in the spatial dimension [34,35]. Accordingly, the high dimensionality of traffic data can be analogized to the multi-channel of image data. Applying the 2-dimensional convolutional neural network (2D CNN) in computer vision to the TFP problem can model the spatial correlation. For instance, Zhang [34] employed a 2Dconv to predict the inflow and outflow of taxis, and Yao [36] also calculated the demand for cabs in urban areas using 2D CNN; Sun [37] performed mutual correlation calculations using a multilayer fully convolutional network to simulate the spatial correlation between current and neighboring sections, local and global scales.

All the above approaches showed comparatively better prediction performance for TFP than traditional model-based approaches. Nevertheless, the complex temporal and spatial characteristics of traffic data will not be limited to a single dimension in practical applications but will be synthesized in a 3-dimensional space [38]. Therefore, comprehensive mining of ST-dependent features is a crucial research component to improve the performance of TFP. Zhang [34,39] proposed a learning method called ST-ResNet to model the closeness, periodicity, and trend of spatiotemporal data using historical flows. Chen [40] and Guo [35] applied 3D convolutional neural networks (3D CNN) to extract the spatiotemporal correlation of data from multi-dimensions. Zhang [41] split the traffic prediction task into node and edge traffic prediction and proposed a multi-task deep learning framework that models the ST interaction from a graph-theoretic perspective. Liu [42] proposed a novel network to learn the dynamic similarity between regions, fully considering the complex spatial dependence and temporal dynamics. Yan [43] dynamically extracted ST features through multiple attention and masked multiple attention mechanisms and determined the significant influential parts of the road network by analyzing the attention weight matrix. Zheng et al. [44] developed a framework that combines CNN and LSTM networks to more effectively extract features of traffic data through an embedding module to fuse external information (e.g., weather, date). For the extraction and modeling of more complicated ST dependencies, Zambrano-Martinez [4] used logistic regression and cluster analysis to predict the geographic distribution of urban traffic behavior, creating a realistic traffic model for a specific target city; to extract the global ST features of traffic information, Fang [45] proposed a neural network method that includes multilayer ST blocks to obtain both global spatial correlation and dynamic temporal features.

In sum, that this may prove fruitful is motivated by the fact that deep learning can obtain more accurate results, not only by eliminating the subjective factors caused by the manual designing of model-driven methods but also by enhancing the nonlinear fitting ability of ST dependencies. A more effective and comprehensive representation of the ST dependencies embedded in traffic data is a crucial part of TFP research to obtain promising prediction results.

### 2.2. Attention for TFP

Attention is essentially an assignment mechanism [46]. The controlling idea is to determine the correlation between them based on the original data, then emphasize important features and realize the reallocation of weights.

Attentional mechanisms enable us to utilize limited attentional resources by filtering out distracting information from the large volume of data, thus significantly reducing information processing errors [42,47,48]. Essentially, the attention mechanism in deep learning is similar to the human visual system in that its purpose is to determine which part of the information may be more valuable for the task. Liu [29] demonstrated the effectiveness of the attention mechanism for TFP by merging three attention modules, channel attention, spatial attention, and location attention, via a deep integration network to achieve adaptive feature refinement. Hao [47] used the sequence-to-sequence model with the attention mechanism to model sequence data of different lengths, and the results have proven that the attention mechanism enhances the ability of the model to capture remote dependencies. Wang [48] proposed a hard attention module that strengthened neuronal memory by learning similar patterns, thus diminishing the accumulation of errors. To reduce error propagation between prediction time steps, Zheng [49] developed a transformed attention module to learn the direct correlation between historical and predicted flows. Do [50] proposed a temporal and spatial attention module for traffic flow prediction, which contributes to extracting the spatiotemporal dependencies between distinct time steps and road networks. Guo [35] designed a spatiotemporal attention module that adaptively adjusts the correlations of graph signal sequences in the temporal and spatial dimensions. Yu [51] used a cross-attention mechanism to fuse ST features to model global information. Jia [52] used a rectified block equipped with the attention mechanism to automatically reweight the measurements for different time intervals. Liu [53] proposed hierarchical attention to extracting features for each time step.

## 3. Problem and Definition

### 3.1. Problem

**Figure 1:**
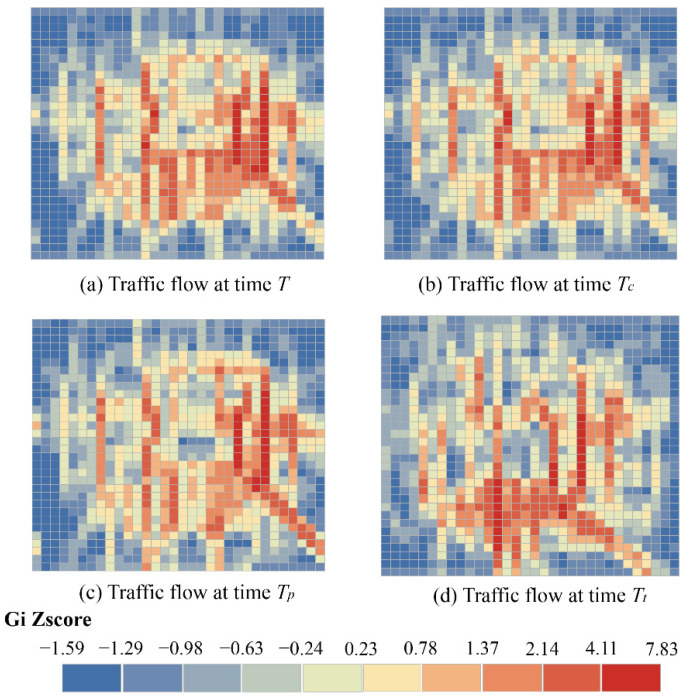
Spatiotemporal aggregation of traffic data.

(1) ST aggregation:

Figure 1 shows the hotspot aggregation characteristics of traffic flow at four moments, *T* represents the traffic flow at the current time and the time interval between $T_{c}$ and *T* is an hour, the time interval between $T_{p}$ and *T* is a complete day, the time interval between $T_{t}$ and *T* is a week. The higher z-score indicates a stronger degree of agglomeration. It can be observed from Figure 1 that the similarity of the flow distribution at *T* with $T_{c}$, $T_{p}$, and $T_{t}$ are decreasing in order from the time perspective. From the spatial perspective, the traffic flow at the four times is not evenly distributed, but concentrated in the city center with significant spatial agglomeration.

Therefore, the general deep learning method that shares parameter weights for all time steps or regions has limitations. Traffic data has agglomeration at different times, which also means that the weights of congested or sparse areas should be different. Given the dynamism of traffic conditions, dynamic adjustment of the weights is also necessary for the prediction task.

**Figure 2:**
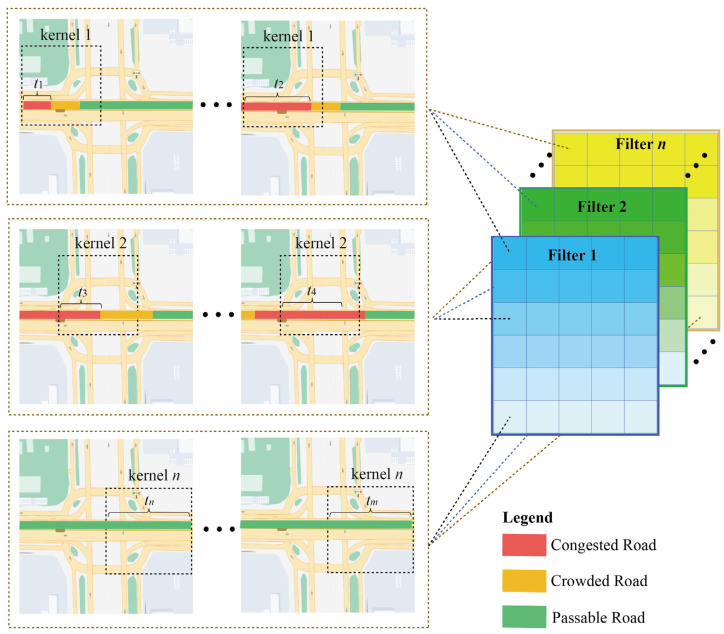
Intrinsic correlation and redundancy of spatiotemporal features.

(2) Intrinsic correlation of the same ST features and redundancy between different ST features:

The ST data obtained at neighboring locations and adjacent time steps are not independent but are interrelated. Taking the traffic congestion situation as an example, traffic congestion does not occur in isolation and generally covers a continuous area and traffic congestion also moves along a 3-dimensional ST domain as time has passed. In this paper, 3D CNN was used to automatically extract the ST features of data, but the extracted ST features remain some problems: In CNN because the kernel is continuously moved to sense the data, the ST feature is extracted by a single filter (a single filter contains *n* kernel) extracted has intrinsic correlation. As shown in Figure 2, the time intervals $t_{1}$ to $t_{2}$ were consecutive. The road is congested at $t_{1}$ and the congestion state propagates eastward along the road network until $t_{2}$, when the congestion state was extended, and all the above information can be sensed by *kernel* 1 in 3-dimensions. For the next consecutive time intervals, $t_{3}$~$t_{4}$, 3D CNN perceives it with *kernel* 2. Although *kernel* 1 senses a different ST domain, the congestion at $t_{3}$ appears not abruptly but was closely related to the traffic state from $t_{1}$ to $t_{2}$. Thus, there is an inherent correlation in the ST features captured by the convolution operation.

In addition, in *kernel n*, the same road was in a passable state in the long-term, and the ST features learned by the kernel of any size were consistent, so there also exists redundant information between multiple ST features. Therefore, it is not reasonable to share weights for all ST features. Learning the intrinsic correlation in ST features, adjusting the weights of the same ST feature to regions, concentrating on information-rich regions, and quantifying the contributions among different ST features to reduce the redundant information were critical issues to improve the prediction performance.

### 3.2. Definition

Data Definition: This paper defined urban traffic data as a 4-dimensional (4D) tensor $X=\left[X_{1},X_{2},\ldots ,X_{T}\right]\in {\mathbb{R}}^{T\times F\times H\times W}$. $X_{T}$ is the OD matrix that counts the outflows or inflows at time T. First, the region was divided into a 2D non-overlapping raster of size H×W according to latitude and longitude, where H and W were the height and width of the regional grid. Secondly, the flow data were stacked to 3D according to F, the total number of types of flow data. Finally, the data were stacked to 4D according to the total number of timestamps T of the flow data.

Problem Definition: The objective of this paper is to build a TFP model: the historical traffic with 3-time intervals of closeness, trend, and period at time t was applied as input $X_{in}=\left[X_{t}^{c},X_{t}^{p},X_{t}^{t}\right]$ to predict multiple types of traffic flow at time t. The summary of the notation can be found in Table 1.

## 4. Methodology

**Figure 3:**
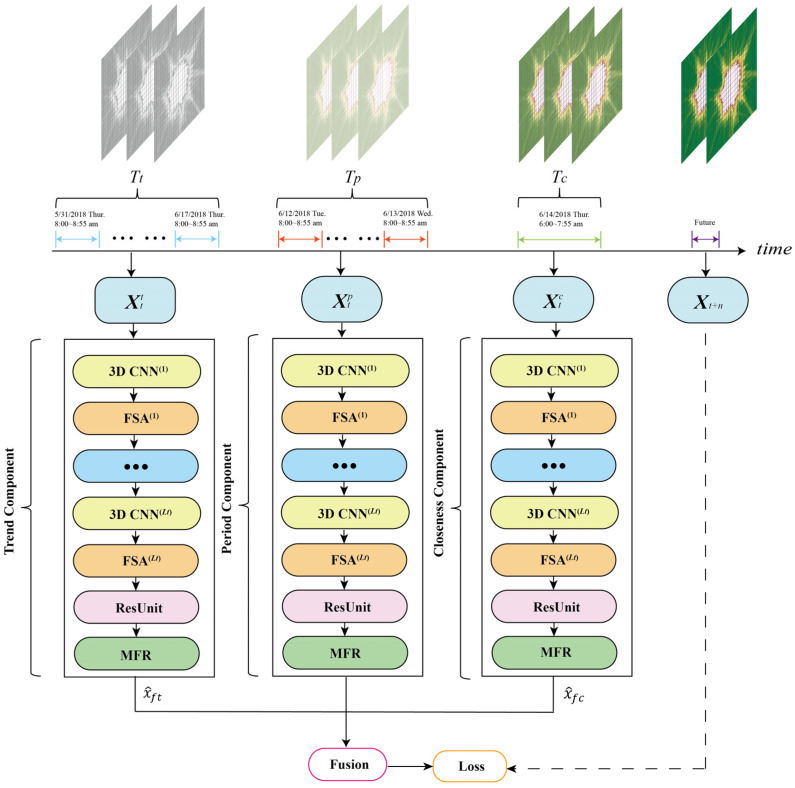
The framework of FASTNN.

Figure 3 shows the framework of FASTNN, this model consists of three basic components of closeness, period, and trend, which intercept three time series of length $T_{c}$, $T_{p}$, and $T_{w}$ along the time as the three component inputs $X_{in}=\{X_{t}^{c},X_{t}^{p},X_{t}^{t}\}$.

1. The closeness component;

$$X_{t}^{c}=\left(X_{t-T_{c}+1},X_{t-T_{c}+2},\ldots ,X_{t}\right)\in {\mathbb{R}}^{I\times J\times F\times T_{c}}$$

2. The period component;

$$X_{t}^{p}=\left(X_{t-T_{p}*q},X_{t-(T_{p}-1)*q},\ldots ,X_{t-q}\right)\in {\mathbb{R}}^{I\times J\times F\times T_{p}}$$

3. The trend component;

$$X_{t}^{t}=\left(X_{t-l*T_{t}*q},X_{t-l*(T_{t}-1)*q},\ldots ,X_{t-l*q}\right)\in {\mathbb{R}}^{I\times J\times F\times T_{t}}$$

where p and q are the period and trend span. $T_{c}$, $T_{p}$, and $T_{t}$ are the time lengths of three components.

The intrinsic structure of each component remains consistent, and these components can extract universal ST dependencies in the data. Taking the closeness component as an example, to extract deep-level spatiotemporal correlations, FASTNN input the historical traffic of closeness into the 3D CNN, and appended the FSA component after the 3D CNN to model the spatiotemporal agglomeration of each feature extracted, thus achieving the dynamic adjustment of the spatial weights. The ST dependencies of the traffic data have been comprehensively modeled after $L_{c}$ replications. To prevent network degradation, FASTNN added residual units after the FSA in the last layer. The ST features processed by residual units still have inherent correlation and redundancy, and these dependencies were modeled by the lightweight MFR proposed in this paper.

### 4.1. 3D Convolutional Neural Network

3D Convolutional neural network (3D CNN) contributes to the model to capture the dependence in the spatiotemporal dimension. Observations obtained at neighboring locations and adjacent time steps are not independent but interrelated and this spatiotemporal correlation can be effectively captured by 3D CNN.

**Figure 4:**
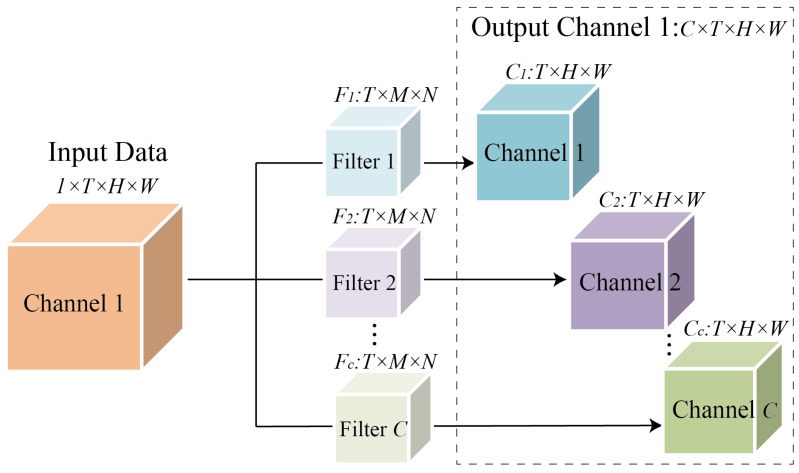
The structure of 3D CNN.

The weights of 3D Convolutional can be expressed as 5-Dimension filters: $F\in {\mathbb{R}}^{C'\times C\times T\times M\times N}$, where $C^{\prime }$ is the number of filters, C is the number of input filters or channels, is the number of input filters or channels, T, M, and N is the temporal length, height, and width of the 3D convolutional filter. Take the closeness component as an example, the input flow was denoted as $X^{l-1}\in {\mathbb{R}}^{C_{l-1}^{'}\times T\times H\times W}$.The calculation of each 3D Convolutional filter $F_{f}\in {\mathbb{R}}^{C\times T\times M\times N},f=1,\ldots ,C^{\prime }$ can be expressed as:

(1) $$\Phi \left(l,i,j\right)=X^{l-1}*F_{f}=\sum_{c=1}^{C}\sum_{t=1}^{T}\sum_{m=1}^{M}\sum_{n=1}^{N}X^{l-1}\left(c,l-t,i-m,j-n\right)F_{f}\left(c,t,m,n\right)$$

where l=1,…,T, m=1,…,M and n=1,…,N. The output flow can be denoted as $X^{l}\in {\mathbb{R}}^{C_{l}^{'}\times L\times M\times N}$. The structure of 3D CNN is shown in Figure 4. Take the input data with the number of channels as 1 as an example, the input data can be expressed as $X\in {\mathbb{R}}^{1\times T\times M\times N}$, after the convolution of filters $F_{f}=\left[F_{1},F_{2},\ldots ,F_{c}\right]$,$\;F_{c}\in {\mathbb{R}}^{C\times T\times M\times N}$, the output channel data $X^{\prime }\in {\mathbb{R}}^{C\times T\times H\times W}$ equal to the number of Filters was obtained. If the input data X contains more than one channel, the number of dimensions of the output data $X^{\prime }$ channels increase accordingly. In the period and trend component, the 3D CNN layer was calculated similarly to the closeness component. After stacking multiple layers of 3D CNN, the critical information of traffic data in the time dimension has been effectively mined.

### 4.2. Filter Spatial Attention

The 3D CNN shows promise in mining information along the ST dimensions but could be difficult to detect the ST agglomeration of traffic data and the agglomeration is dynamically changing, it is also difficult to adaptively adjust the region weight. Consequently, this paper used the filter spatial attention (FSA) module to dynamically adjust the intensity of ST agglomeration based on the input data. To compare the model performance of different attention mechanisms, this paper also compared the experimental performances of two different mechanisms, namely, multi-headed attention, self-attention, and the FSA proposed in this paper. The equation for calculating FSA was as follows:


(2) $$S_{k}=V_{s}\cdot \sigma (Q_{s}\cdot K_{s}+b_{s})=V_{s}\cdot \sigma (\left(X^{\left(r-1\right)}W_{1}\right)W_{2}{\left(W_{3}X^{\left(r-1\right)}\right)}^{T}+b_{s})$$

(3) $$S=\frac{\exp\left(S_{k}\right)}{\sum_{j=1}^{N}\exp\left(S_{k}\right)}$$

In Equation (2), $V_{s},b_{s}\in R^{N\times N}$, $W_{1}$, $W_{2}$ and $W_{3}$ were learn-able parameters, which is trained using gradient descent, $W_{1}\in {\mathbb{R}}^{T_{r-1}\times 1}$, $W_{2}\in R^{C_{r-1}\times T_{r-1}}$, $W_{3}\in {\mathbb{R}}^{C_{r-1}\times 1}$ and $X^{\left(r-1\right)}=\left(X_{1},X_{2},\ldots ,X_{T_{r-1}}\right)\in {\mathbb{R}}^{N\times C_{r-1}\times T_{r-1}}$, σ is the sigmoid function and $X^{\left(r-1\right)}$ is the output of rth 3D CNN.$\;T_{r-1}$ is the output time length of (r−1)th 3D CNN and $C_{r-1}$ is the output filter length of (r−1)th 3D CNN. N=H×W is the total number of regional grids.

**Figure 5:**
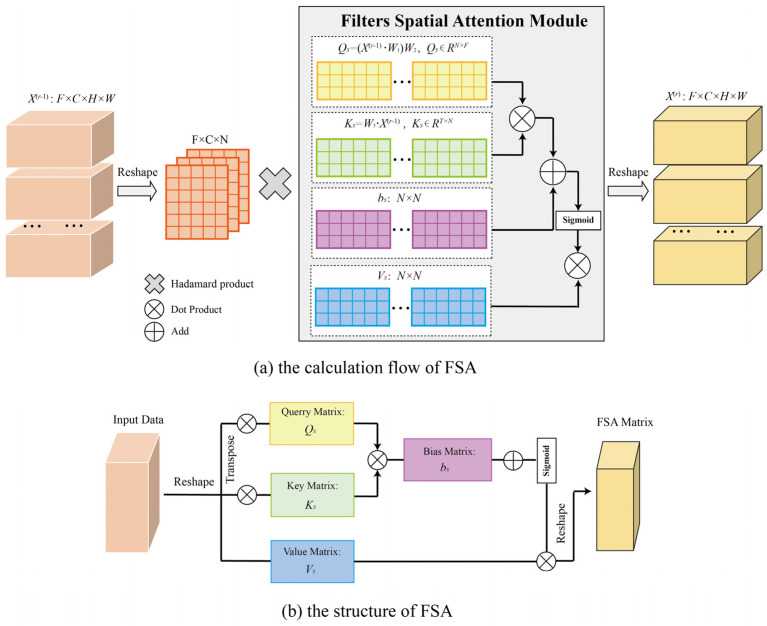
The calculation flow and structure of FSA.

The calculation flow and structure of FSA were presented in Figure 5. In Figure 5a, take the closeness component as an example, the 3D CNN input of the lth layer is ${\hat{x}}_{c}^{l}$ and ${\hat{x}}_{c}^{l}={\hat{x}}_{c}^{l-1}\circ S$ was used to model ST agglomeration, which ∘ denotes the Hadamard product and S is the spatial weight matrix calculated by the FSA module. When l=1, ${\hat{x}}_{c}^{l}\in {\mathbb{R}}^{F\times T_{c}\times H\times W}$, $T_{c}$ is the input time length of the closeness component. When l+n,n≥1, to realize the fusion with the FSA module, the input ${\hat{X}}_{c}^{\left(l+n\right)}\in {\mathbb{R}}^{F\times C_{\left(l+n-1\right)}\times H\times W}$ was reshaped as ${\hat{X}}_{c}^{\left(l+n\right)}\in {\mathbb{R}}^{F\times C_{\left(l+n-1\right)}\times N}$, where $C_{\left(l+n-1\right)}$ was the filter number of the (l+n−1)th 3D CNN layer. After multiplying the output ${\hat{X}}_{c}^{\left(l+n\right)}$ with S can obtained the input ${\hat{x}}_{c}^{\left(l+n+1\right)}$ of (l+n+1)th 3D CNN, the input was then reshaped back to ${\hat{x}}_{c}^{\left(l+n+1\right)}\in {\mathbb{R}}^{F\times C_{\left(l+n\right)}\times H\times W}$. In the period and trend components, the calculation was completely consistent.

Figure 5b shows the structure of FSA. The structure of FSA is referenced to the general attention mechanism, in which the feature matrices are calculated by $Q_{s}$, $K_{s}$, and $V_{s}$. The difference with the general attention is the difference between the calculation method and data dimensionality: The attention uses the method of vector intersection to determine the similarity, while FSA uses multi-dimensional learning parameters and more dot product operations to determine the similarity more comprehensively.

### 4.3. Residual Unit

After stacking multiple layers of 3D CNN and modules of FSA, the dependencies of traffic data in ST dimensions have been comprehensively mined. As the number of neural network layers deepens, the training of the network becomes more difficult and even leads to performance degradation in the network.

As the depth of the neural network layers deepens, the training of the network could become more difficult and result in even degradation of the network performance. To alleviate the degradation phenomenon caused by the deepening of neural network layers, the residual unit proposed by He [54] was employed in this paper to guarantee the training performance of the model. In this paper, $L_{r}$ residual units were stacked after the last layer of 3D CNN, which were calculated as follows:

(4) $${\hat{x}}^{\left(L_{c}+l\right)}={\hat{x}}^{\left(L_{c}+l-1\right)}+F\left(X_{c}^{\left(L_{c}+l-1\right)};\theta_{c}^{l}\right),l=1,\ldots ,L_{r}$$

In Equation (4), $\theta_{c}^{l}$ is the set of all learnable parameters in the lth residual unit. ${\hat{x}}^{\left(L_{c}+l\right)}$ is the output of ${\left(L_{c}\right)}^{th}$ residual unit and ${\hat{x}}^{\left(L_{c}+l-1\right)}$ is the input. When $L_{c}=1$, to make the residual unit fuse with the FSA module output, the input of $\left(L_{c}\right)$th the residual unit was reconstructed as ${\hat{x}}^{\left(L_{c}\right)}\in {\mathbb{R}}^{C_{l}\times H\times W}$, and $C_{l}$ is the feature number of the FSA output of the last module.

### 4.4. Matrix Factorization Based Resample Module

Quantifying and adjusting the weights of regions for the same ST feature, enhancing the focus on information-rich regions, and reducing the redundant information in different ST features was a critical aspect to improve the performance of the TFP model. However, modeling spatiotemporal features using a single set of parameter weights cannot model the nonlinear relationships among multiple spatiotemporal features. It is necessary to enable each filter to correspond to a separate prediction network. However, independent training of each filter’s prediction network introduces new problems:

- Independent training cannot model the correlation between multiple ST features, nor can it eliminate redundant ST features [35,55,56];
- Direct training using fully-connected layers introduces excessive training parameters that can lead to difficult optimization or overfitting of the model.

**Figure 6:**
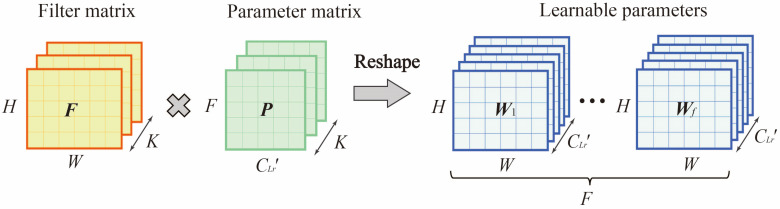
Matrix decomposition of learnable parameters in MFR.

To address this problem, the matrix factorization-based resample module (MFR) was proposed in this paper. This module can automatically learn the contribution of each region in the same spatiotemporal features and the correlation between different spatiotemporal features, thus improving the model representation and prediction capability. The input to the MFR module was $x_{c}^{i}\in R{\mathbb{R}}^{F\times C_{Lr}^{'}\times H\times W}$. The output after training was the ${\hat{x}}_{c}^{R}\in {\mathbb{R}}^{F\times H\times W}$. $F_{R}$ was the set of learnable parameters, $F_{R}=\left[f_{1},\ldots ,f_{C^{R}}\right],f_{i}\in R^{F\times H\times W}$, where $C^{R}$ was the number of ST features (i.e filters). As Figure 6 shown, this paper used a Filter Matrix $F\in {\mathbb{R}}^{H\times W\times K}$ and a Parameter Matrix $P\in R^{C_{Lr}^{'}\times F\times K}$ to approximate $F_{R}\in R{\mathbb{R}}^{C_{Lr}^{'}\times F\times H\times W}$, where K is a constant less than $C_{Lr}^{'}\times F$.

(5) $$F_{R}=reshape\left(W{\left(C\right)}^{T}\right)$$

(6) $$X_{c}=F_{R}\cdot X_{c}^{L_{r}}+b_{i}$$

In Equation (6),$\;b_{i}$ represents the bias term of the ith ST features, $b=\left[b_{1},\ldots ,b_{i}\right]\in {\mathbb{R}}^{F\times W\times K}$, also calculated by the matrix factorization.

### 4.5. Fusion Component

When fusing the outputs of components closeness, period, and trend, the fully-connected neural network (FNN) was used to automatically learn the importance of the three types of outputs. The output of the closeness component, the period component, and the trend component can be expressed as [${\hat{x}}_{fc},{\hat{x}}_{fp},{\hat{x}}_{ft}$]. The fusion component can be expressed as follows:

(7) $$X_{t}^{'}=W_{t}\circ {\hat{x}}_{ft}+W_{p}\circ {\hat{x}}_{fp}+W_{t}\circ {\hat{x}}_{ft}$$

where $W_{t},W_{c}\;and\;W_{p}$ is the learnable parameter, ∘ representing the Hadamard product.

### 4.6. Loss Function

The model was trained by minimizing the loss function, which is defined as the mean root error (MSE) between the true traffic raster values and the predicted values. MSE was used for the reason that it is continuously derivable, which facilitates the use of gradient descent algorithms and also facilitates the convergence of the function. The formula for MSE is as follows:

(8) $$ℒ\left(\theta \right)=∥X_{t}^{'}-\hat{X_{t}}{∥}_{2}^{2}$$

where θ is the learnable parameters, $X_{t}^{'}$ is the predicted traffic flow at time t and $\hat{X_{t}}$ is the true traffic flow at time t.

## 5. Experiments

The main objective of the urban traffic flow predicting task was to build an accurate model to predict multiple flows for a specific demand in each time and region of the city. This paper demonstrated the application of the FASTNN to an urban traffic flow forecasting task on two large-scale datasets (TaxiBJ and BikeNYC). The results of the paper were intended to answer the following questions:

1. How does the FASTNN proposed in this paper perform compared to the baselines?
2. What is the performance of the FASTNN variants with different modules?
3. How effective are the FSA module and the MFR module proposed in this paper?
4. Why are FSA and MFR effective?

### 5.1. Dataset

In this paper, two traffic flow datasets, TaxiBJ and BikeNYC, were used to verify the performance of the FASTNN, and the details of the two datasets were shown in Table 2. The common feature of both datasets is that the area was transformed into an H×W grid, and the traffic flow data was transformed into raster data with 2 channels. The two channels were traffic inflow and outflow.

- TaxiBJ dataset is crowd flow data obtained from GPS trajectory data of Beijing cabs, which contains four-time intervals: 1 July 2013, to 30 October 2013; 1 March 2014 to 30 June 2014; 1 March 2015, to 30 June 2015; and 1 November 2015, to 10 April 2016. This dataset firstly divides the main urban area of Beijing into 32 × 32 grid areas, and secondly counts the origin and destination points of each vehicle trajectory in the above four time periods according to the 0.5 h interval. Because the dataset has ST continuity, the dataset can detect all traffic conditions under a specific region;
- BikeNYC dataset is obtained from 1 April to 30 September 2014, New York City Bicycle System [39]. This dataset divides the main city of New York into a 16 × 8 grid, and counts the inflow and outflow of crowds within the area at one-hour time intervals, with a total number of time timestamps of 4392. This dataset is based on the 2014 NYC Bike system bike-sharing trip data and counts the traffic flow within the 16 × 8 grid according to the bike-sharing orders in each area, by latitude and longitude.

### 5.2. Baselines

In this paper, the FASTNN was compared with the following baselines:

- History Average Model (HA): The predicted flow of the model is the average of the recent historical traffic data at the corresponding time;
- Autoregressive Integrated Moving Average Model (ARIMA): ARIMA regards the data series of the prediction object over time as a random sequence, and uses a certain mathematical model to describe this sequence approximately;
- Support Vector Regression (SVR): SVR utilizes linear support vector machines for regression tasks, and the central idea of the model is to find a regression plane such that all the data in a set are closest to that plane;
- Long Short-Term Memory (LSTM): LSTM is a neural network with the ability to remember long and short-term information, consisting of a unit, input gates, output gates, and forgetting gates, for solving the problem of long-term dependencies;
- Gated Recurrent Unit (GRU): GRU [57] is a variant of LSTM. A gating mechanism is used to control the input, memory, and other information, while making predictions at the current time step;
- ConvLSTM: The convolution mechanism [58], which can extract spatial features, is added to the LSTM network, which can extract temporal features and can capture ST relationships;
- ST-ResNet: Spatiotemporal residual network [39], which utilizes three residual neural network components to model the temporal closeness, period, and trend properties of urban flows;
- ST3Dnet: An end-to-end deep learning model [18], ST3Dnet uses the 3D CNN and recalibration module to model the local and global dependencies.

### 5.3. Evaluation Metrics

To better evaluate the performance improvement of the FASTNN, this paper used the following two metrics for evaluation.

Root Mean Squared Error (RMSE):

(9) $$\mathrm{RMSE}=\sqrt{\frac{1}{T}\sum_{i}^{t}{\left({\hat{y}}_{i}-y_{i}\right)}^{2}}$$

Mean Absolute Error (MAE):

(10) $$\;\mathrm{MAE}=\frac{1}{T}\sum_{i}^{t}\left|{\hat{y}}_{i}-y_{i}\right|$$

In Equations (9) and (10), where ${\hat{y}}_{i}$ is the predicted traffic flow, $y_{i}$ is the real traffic flow in the region, and T is the total number of time intervals, which also is the total number of samples.

### 5.4. Model Training

The FASTNN was constructed based on the TensorFlow framework and was trained and tested on an Ubuntu 16.04 server with a single graphics card (NVIDIA GTX 3060Ti). In the model training, the batch size was set to 16, the learning rate was set to 0.002, and the early stopping strategy was used to prevent overfitting. The two datasets were divided into respective training dataset, validation dataset, and test dataset in time order. These two datasets did not overlap with each other and were divided in a proportion of 8:1:1 on the time series.

**Figure 7:**
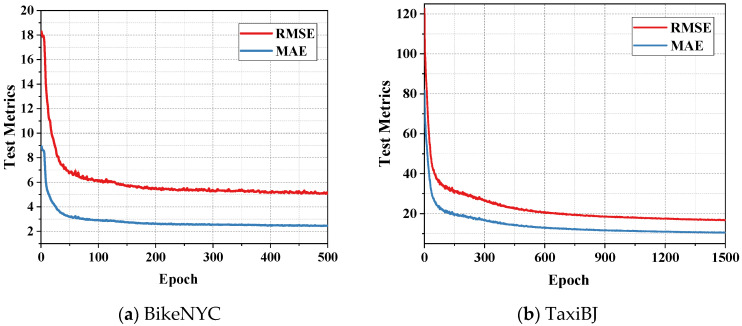
The testing metrics on two datasets.

The adaptive moment estimation (Adam) optimization algorithm was used in the model for end-to-end gradient descent training. The RMSE and MAE curves during model training were shown in Figure 7. It can be observed that the FASTNN was properly trained and not overfitted on the two large-scale traffic datasets.

### 5.5. Performance Comparison with Baselines (Q1)

Table 3 presents the variation in the performance of the FASTNN and other baselines on the two datasets. For the FASTNN and all baselines, this paper used different random seeds for training, tested three times, and record the experimental results and error margin in the format of “mean ± error margin”. From Table 3, the following conclusions can be derived.

Compared to traditional time series analysis methods and machine learning methods (e.g., HA, ARIMA, and SVR), deep learning-based baselines have better predictive performance for all evaluation metrics. These findings are understandable because machine learning methods have limited capability to model nonlinear ST features. Moreover, for LSTM and CNN, which can only model temporal or spatial features from a single dimension, models, such as ConvLSTM and ST-ResNet, which can model ST dependencies from multiple dimensions, evidently achieve better performance.

In the TFP, the FASTNN achieves better prediction performance than existing baseline approaches. Compared to the best performance in traditional baselines (i.e., HA, ARIMA, and SVR) for the BikeNYC and TaxiBJ datasets, the FASTNN achieved relative improvements of 54.26% and 37.45% (RMSE), while MAE achieved a relative improvement of 61.08% and 43.05%. Compared to the best performance in deep learning-based baselines, the FASTNN achieved relative improvements of 22.94% and 9.86% (RMSE) in BikeNYC and TaxiBJ datasets. Similar improvement results were presented in the comparison of MAE metrics, and the improvement of MAE was 32.04% and 5.15%.

The architectural modules of FASTNN contribute to these improvements. Other baseline methods disregard the spatial agglomeration of traffic flow at different time intervals and use a weight-sharing training strategy for all regions. The FASTNN, on the contrary, incorporated the FSA module, which can dynamically adjust the region weights in each training step, and effectively distinguishes the traffic agglomeration regions from the sparse regions. Moreover, based on the concept of intrinsic correlation of the same ST features and redundancy between different ST features proposed in this paper, the FASTNN used the MFR module to automatically learn the intrinsic correlations in the same ST features and calculate their spatial weights. This module also can enhance the importance of information-rich features and reduce the impact of redundant information features, thus improving the prediction performance of the model.

### 5.6. Evaluations on Variants of the Module (Q2)

To investigate what is the performance of FASTNN variants with different modules, the FSA and MFR modules were varied and replaced in the FASTNN. The FSA module was based on the attention mechanism; thus, this paper evaluated the performance of two general variants, the multi-headed attention mechanism (MA), and the self-attention mechanism (SA) [46]. For the MFR module, which is capable of automatic learning intrinsic correlation and disregarding redundant information, this paper has compared it using the forward neural network (FNN) and the adding layer. Detailed variant model descriptions were shown as follows:

- STNN: This model has removed all FSA modules and MFR modules from the FASTNN, remaining the components of 3D CNN and the residual unit;
- FASTNN-MA: This model has replaced the FSA module in the FASTNN with the MA;
- FASTNN-SA: This model has replaced the FSA module in the FASTNN with the SA;
- FASTNN-FNN: This model has replaced the MFR module in the FASTNN with the FNN;
- FASTNN-add: The FASTNN-add model has replaced the MFR module in the FASTNN with the adding layer, the adding layer can sum the ST features by filters.

Table 4 shows the performance of FASTNN compared with other variants of the model. It can be observed that FASTNN proposed in this paper achieves the best performance compared to all variants.

In the attention variants, a possible explanation for this is that FASTNN-MA and FASTNN-SA not only required reconstructing the learnable parameters to sequence length but also relied on manually setting the sequence length, which resulted in the possibility of dropping critical information for a shorter length during the computation. Longer sequence length, on the other hand, will increase the number of parameters in the model and result in overfitting problems in the model. For example, the FASTNN-MA model outperforms FASTNN-SA in the TaxiBJ dataset, which has a larger volume of data, while the opposite prediction performance was observed in the BikeNYC dataset, which has a smaller volume. Meanwhile, the MAE metric of FASTN-MA is slightly better than that of FASTNN in the TaxiBJ dataset, a possible explanation for this is that FASTNN-MA produced outliers in the prediction task of the TaxiBJ dataset with a larger data volume, which was detected by RMSE but not by MAE due to the different metric calculation.

In the MFR variant, the performance of FNN was better than that of the adding layer, which indicates that each ST feature contains information of different importance to the model. However, the direct calculation of contribution using FNN will ignore the intrinsic correlation in the same ST feature and introduce redundant information between different ST features, which results in the reduction of model accuracy.

### 5.7. Evaluations on Ablation Analysis (Q3)

To quantify the effectiveness of the FSA module and MFR module proposed in this paper, the following ablation analysis was conducted. This paper evaluated the prediction performance of the original model, the model without the FSA module (FASTNN-without FSA), and the model without the MFR module (FASTNN-without MFR), on the datasets using two metrics.

**Figure 8:**
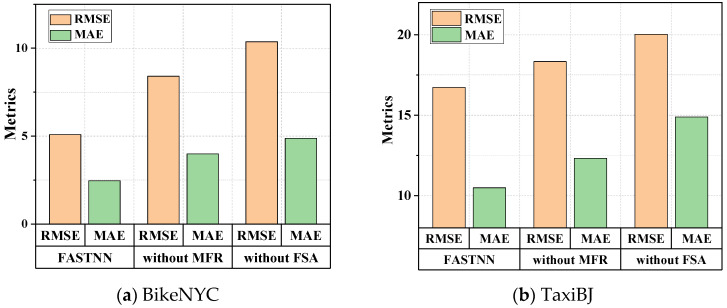
Comparison of ablation experiments on two datasets.

As shown in Figure 8, the accuracy of FASTNN-without FSA was consistently lower than that of the FASTNN given the lack of display modeling of the ST aggregation. Simultaneously, the accuracy of FASTNN-without FSA was additionally lower than that of FASTNN-without MFR, indicating a greater degree of importance for ST agglomerative deep mining in the TFP, and the quantification of the intrinsic correlation and redundancy brought the performance improvement less than its obvious effect.

**Figure 9:**
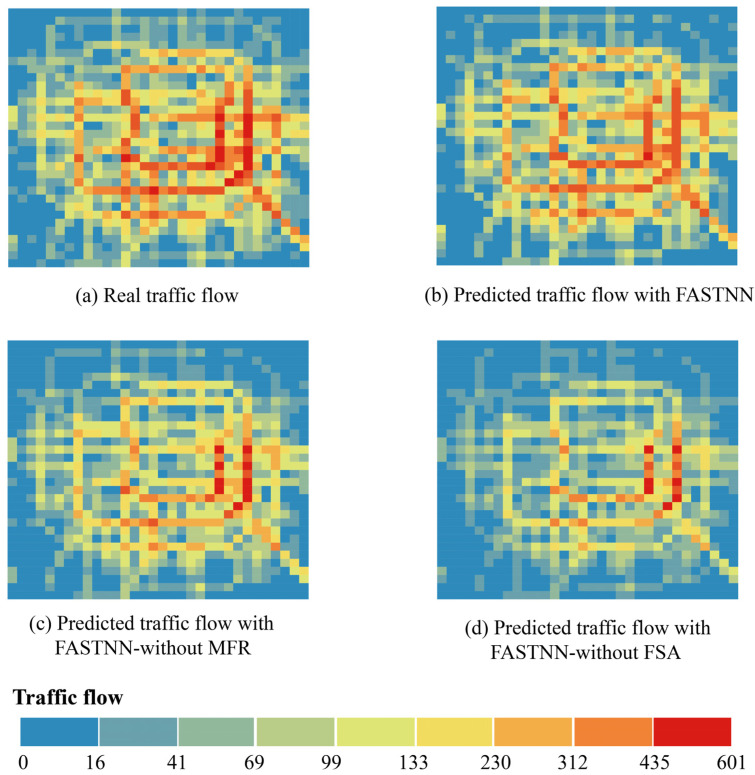
Real traffic flow and model prediction results.

Figure 9 visualized the real traffic flow and the prediction results of each model. Among them, Figure 9a showed the visualization results of the original real traffic flow at moment t+1, and Figure 9b–d show the traffic flow prediction results of FASTNN, FASTNN-without MFR, and FASTNN-without FSA at moment t+1. The prediction result of FASTNN was the closest to the real traffic flow, which restores the real state of traffic flow to the greatest extent, and the prediction result of FASTNN-without MFR is secondary. The prediction of FASTNN-without FSA is underperforming, and the congestion characteristics in the center and the traffic flow in the edge part are not detectable efficiently.

### 5.8. Effective of the Module (Q4)

**Figure 10:**
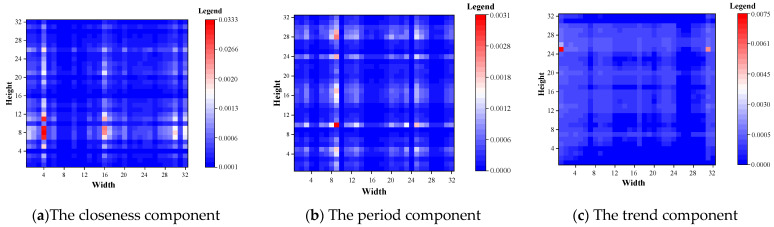
Visualization of FSA module for TaxiBJ dataset.

This paper visualized the FSA weight matrix of the output of the FSA module of the last layer of the three components of closeness period and trend using the TaxiBJ dataset as an example. As shown in Figure 10, the weights of all regions were greater than 0, indicating that all regions have a positive effect on the TFP. The closeness component has the maximum weight with a mean value of 0.000455 and the period component has the minimum weight with a mean value of 0.000301. In addition, the distribution pattern of the hotspot of the closeness component was similar to that of the period component, which indicated that the closer the input historical time is to the predicted time, the greater the contribution to the prediction.

To visualize the effectiveness of the MFR module, the weight matrices of the outflows and inflows of the three components closeness, period, and trend in the MFR layer were visualized on its 32 × 32 grid using the TaxiBJ dataset as an example.

**Figure 11:**
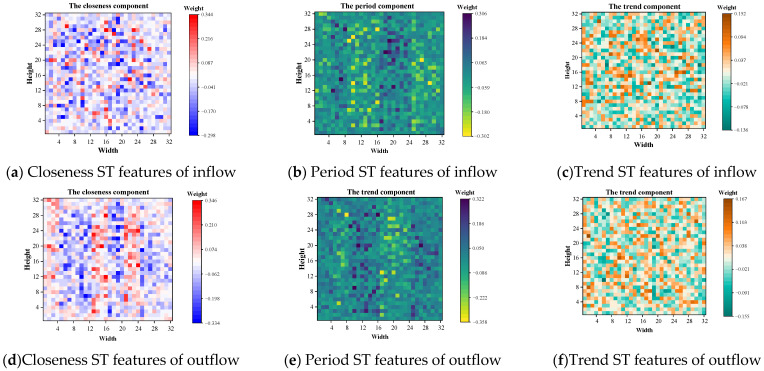
Visualization of MFR module for TaxiBJ dataset.

The results were shown in Figure 11. In each weight matrix, the value of grid (i,j) indicated the MFR module weight of the corresponding ST feature to the (i,j) region, which has modeled the intrinsic correlation of each ST feature and the redundancy between all ST features. It can be observed that the same ST features have different contributions to each region, as in Figure 11a, each region has different weight values, which also represents the successful modeling of the intrinsic correlation. Simultaneously, different ST features also have different contributions to the same region, as in Figure 11a–c, the weight values of the same region were different in different components, which represents the successful modeling of redundancy for different ST features.

## 6. Conclusions

Traffic flow prediction is a key input to intelligent transportation systems, intending to predict short-term or long-term traffic flow based on historical traffic data. Accurate TFP for the region can pre-allocate the required vehicles to areas with high travel demand, which not only reduces passenger waiting time and improves travel service quality but also provide references for implementing traffic management strategies, developing travel route planning, and public transportation risk assessment.

The starting point of this paper is to build an accurate deep learning model for traffic flow prediction. The motivation of this paper is to model the two key problems of spatial-temporal aggregation in traffic data and intrinsic correlation or redundancy of the spatialtemporal features and thus implement the deep mining of the spatiotemporal dependence of traffic data to improve the prediction accuracy. To solve mentioned problem, this paper proposed a novel deep learning model, named filter attention-based spatiotemporal neural network. This model used the filter spatial attention module, which can implement the dynamic adjustment of spatial weights of ST features under different times and regions. This model also constructed a lightweight matrix factorization-based resample module that models the intrinsic correlation in the ST feature, which also enhances the concentration of the model to information-rich ST features and reduces redundancy among different ST features. Meanwhile, this paper employed three types of historical traffic data-closeness, period, and trend- and 3D-convolutional neural networks to mine generic spatiotemporal dependencies. The specific experimental conclusions were as follows:

(1) In the comparison of the baseline models, the deep learning-based baselines have better predictive performance than the traditional baselines, which indicates that deep learning-based baselines are capable of eliminating the subjective factors caused by the artificial design compared to traditional baselines and also have enhanced spatiotemporal dependent nonlinear fitting capability;
(2) The performance of the FASTNN was evaluated using two large-scale real datasets, and the results indicate that the FASTNN achieves more accurate predictions than the existing baselines, and the performance of FASTNN improves by 22.94% and 9.86% (RMSE) on the BikeNYC and TaxiBJ datasets compared to the baseline with optimal performance. Simultaneously, the same predicted performance results also appear in the variant experiments;
(3) In the ablation analysis, the FASTNN model with FSA predicted better performance than the model with MFR, indicating that modeling of spatiotemporal aggregation is more critical than the modeling of intrinsic correlation and redundancy of spatiotemporal features.

It is noteworthy that the FASTNN can run without extensive external features and achieve better results. This suggests that modeling the spatiotemporal aggregation of traffic data and quantifying the intrinsic correlation and redundancy between ST features can contribute positively to the extraction of nonlinear spatiotemporal dependencies. The FASTNN proposed in this paper can provide reliable traffic guidance information to intelligent transportation systems. In future work, we consider incorporating the extensive multi-source data (e.g., transit, bike) into the traffic flow prediction to mine and model the interactions and correlations between spatiotemporal data. Meanwhile, the incorporation of external traffic information, such as road networks and traffic lights, is also an important direction for TFP to consider. Limited by the availability of data, external features were not considered here in this paper.

## Figures and Tables

**Table 1:** Summary of Notation.

Notations	Description
T	The time length of the data
F	Data channels
H	The height of regions
W	The width of regions
N	The gird number of regions
$X_{t}^{c}$	Input data of c time intervals adjacent to time interval t
$X_{t}^{p}$	The adjacent data of p-day for the same time intervals as t.
$X_{t}^{t}$	The adjacent data of t-week for the same time intervals as t.
$X_{t}^{'}$	Final prediction at time t.
$C_{r}$	Number of ST features of the $r^{th}$ layer network
$T_{r}$	Data time length of $r^{th}$ layer network

**Table 2:** Details of the dataset.

Dataset	TaxiBJ	BikeNYC
City	Beijing	New York
Time-span	7/1/2013–10/30/2013 3/1/2014–6/30/2014 3/1/2015–6/30/2015 11/1/2015–4/10/2016	4/1/2014–9/30/2014
Time interval	30 min	1 h
Map size	32 × 32	16 × 8
Number of timestamps	22,459	4392

**Table 3:** Comparison of performance under different baselines. (Note: Bold represents the best performance).

Baselines	BikeNYC		TaxiBJ	
	RMSE	MAE	RMSE	MAE
HA	12.56 ± 0.00	6.35 ± 0.00	53.21 ± 0.00	26.70 ± 0.00
ARIMA	11.56 ± 0.00	6.79 ± 0.00	28.65 ± 0.00	19.32 ± 0.00
SVR	11.02 ± 0.01	6.32 ± 0.07	26.75 ± 0.15	18.42 ± 0.09
LSTM	9.12 ± 0.69	5.31 ± 0.42	24.34 ± 0.50	16.76 ± 0.56
CNN	9.04 ± 0.57	4.98 ± 0.11	26.58 ± 0.23	14.02 ± 0.12
ConvLSTM	8.23 ± 2.49	4.36 ± 1.27	23.42 ± 1.36	13.24 ± 3.11
ST-ResNet	7.03 ± 0.72	3.94 ± 1.05	19.21 ± 0.56	12.97 ± 2.01
ST3Dnet	6.54 ± 1.03	3.62 ± 0.74	18.56 ± 0.59	11.06 ± 1.56
FASTNN [ours]	**5.04 ± 0.68**	**2.46 ± 0.58**	**16.73 ± 0.36**	**10.49 ± 0.91**

**Table 4:** Comparison of performance under different variants. (Note: Bold represents the best performance).

Variant	TaxiBJ		BikeNYC	
	RMSE	MAE	RMSE	MAE
FASTNN	**16.73 ± 0.36**	10.49 ± 0.91	**5.04 ± 0.68**	**2.46 ± 0.58**
STNN	23.56 ± 0.69	13.36 ± 0.33	9.94 ± 0.50	5.02 ± 0.23
FASTNN-SA	21.18 ± 0.53	11.47 ± 0.47	8.14 ± 0.27	4.26 ± 0.16
FASTNN-MA	20.87 ± 0.69	**10.13 ± 0.35**	9.04 ± 0.48	4.88 ± 0.20
FASTNN-FNN	17.50 ± 0.35	10.93 ± 0.16	5.93 ± 0.25	3.56 ± 0.11
FASTNN-add	18.87 ± 0.15	11.51 ± 0.04	7.83 ± 0.23	3.98 ± 0.09

## Data Availability

Not applicable.
